# The value of computed tomography texture analysis in identifying chronic subdural hematoma patients with a good response to polytherapy

**DOI:** 10.1038/s41598-024-53376-7

**Published:** 2024-02-12

**Authors:** Zhuang Sha, Di Wu, Shiying Dong, Tao Liu, Chenrui Wu, Chuanxiang Lv, Mingqi Liu, Weiwei Jiang, Jiangyuan Yuan, Meng Nie, Chuang Gao, Feng Liu, Xinjie Zhang, Rongcai Jiang

**Affiliations:** 1 Department of Neurosurgery, Tianjin Medical University General Hospital, Tianjin, China; 2 Tianjin Neurological Institute, Key Laboratory of Post-Neuroinjury, Neuro-Repair, and Regeneration in the Central Nervous System, Tianjin Medical University General Hospital, Ministry of Education, Tianjin, China; 3 Department of Neurosurgery, The First Hospital of Jilin University, Changchun, China; 4 Department of Radiology and Tianjin Key Laboratory of Functional Imaging, Tianjin Medical University General Hospital, Tianjin, China; 5 State Key Laboratory of Experimental Hematology, Tianjin Medical University General Hospital, Tianjin, China

**Keywords:** Brain injuries, Outcomes research

## Abstract

This study aimed to investigate the predictive factors of therapeutic efficacy for chronic subdural hematoma (CSDH) patients receiving atorvastatin combined with dexamethasone therapy by using clinical imaging characteristics in conjunction with computed tomography (CT) texture analysis (CTTA). Clinical imaging characteristics and CT texture parameters at admission were retrospectively investigated in 141 CSDH patients who received atorvastatin combined with dexamethasone therapy from June 2019 to December 2022. The patients were divided into a training set (n = 81) and a validation set (n = 60). Patients in the training data were divided into two groups based on the effectiveness of the treatment. Univariate and multivariate analyses were performed to assess the potential factors that could indicate the prognosis of CSDH patients in the training set. The receiver operating characteristic (ROC) curve was used to analyze the predictive efficacy of the significant factors in predicting the prognosis of CSDH patients and was validated using a validation set. The multivariate analysis showed that the hematoma density to brain parenchyma density ratio, singal min (minimum) and singal standard deviation of the pixel distribution histogram, and inhomogeneity were independent predictors for the prognosis of CSDH patients based on atorvastatin and dexamethasone therapy. The area under the ROC curve between the two groups was between 0.716 and 0.806. As determined by significant factors, the validation's accuracy range was 0.816 to 0.952. Clinical imaging characteristics in conjunction with CTTA could aid in distinguishing patients with CSDH who responded well to atorvastatin combined with dexamethasone.

## Introduction

Chronic subdural hematoma (CSDH) is very common in neurosurgery^1^, mainly due to the accumulation of hemorrhage in the subdural space, and it is most common in middle-aged and elderly people. When the hematoma is large, there may be symptoms such as brain tissue compression, a progressive increase in intracranial pressure, and cerebral circulation obstruction, which is prone to brain herniation and threatens the patient’s life and safety. Surgery with subdural drainage is the mainstay treatment^1–6^. Due to relevant surgical complications, a recurrence risk of up to 30%, and the increased mortality in this vulnerable patient population^7–10^, safe and effective nonsurgical treatments are highly desirable. Atorvastatin (ATO), a 3-hydroxy3-methylglutaryl-coenzyme A (HMG-CoA) reductase synthetic competitive inhibitor, has been found to promote angiogenesis and reduce inflammation, both of which are associated with the formation of CSDH^11–14^. Furthermore, several clinical studies have shown that ATO promotes hematoma resorption in CSDH patients without surgery^15–18^. Recently, some studies have shown that atorvastatin combined with dexamethasone (DXM) promotes hematoma absorption and protects endothelial function in an optimized rat model and patients with chronic subdural hematoma^19–21^. However, some patients could not obtain benefits from the treatment of ATO combined with DXM, and it would be unsafe and rewardless to use polytherapy in patients without self-absorption ability. It is important to look for indicators of the prognosis of CSDH patients receiving polytherapy.

Computed tomography (CT) scanning has become the examination of choice for patients with CSDH. CT images contain a large amount of texture information, including the gray level distribution, spatial relationship, and pixel characteristics. In recent years, texture analysis has emerged as a quantitative analysis method in clinical research^22–24^. Texture features play a valuable role in classifying and staging diseases that are challenging to differentiate using traditional CT and magnetic resonance imaging (MRI) methods. Moreover, texture analysis eliminates the reliance on subjective clinical judgment and can be applied to analyze diverse types of images. To the best of our knowledge, there have been no relevant studies on predictive imaging markers for the prognosis of CSDH patients receiving polytherapy using computed tomography texture analysis. This study aimed to explore imaging parameters to distinguish CSDH patients who responded well to the polytherapy via clinical imaging characteristics in conjunction with computed tomography texture analysis.

## Methods

### Patients

All consecutive patients with chronic subdural hematoma receiving polytherapy at the Department of Neurosurgery, Tianjin Medical University General Hospital, between June 2019 and December 2022 were retrospectively reviewed. The following inclusion criteria were used: (a) patients receiving polytherapy (ATO combined with DXM); (b) head CT images in Digital Imaging and Communications in Medicine (DICOM) format before treatments were available; (c) Markwalder grading scale and Glasgow Coma Scale (MGS–GCS) grade of 0–2; and (d) patients who had received a follow-up period of at least 12 weeks. The exclusion criteria for the analysis included (a) CSDH caused by cancer, hemopathy, or other known serious comorbidities; (b) CSDH patients with other intracranial lesions such as brain contusion and laceration, subarachnoid hemorrhage, cerebral infarction, or cerebral edema; (c) CT images with low quality such as artifacts and incomplete coverage of CSDH in the scanning range; and (d) recurrence after CSDH surgery.

### Treatment and follow-up

Chronic subdural hematoma patients diagnosed by head CT scan were given 20 mg of oral atorvastatin (Pfizer Inc.) and low-dose dexamethasone (Tianyao Pharmaceutical Co. Ltd.) every day for five consecutive weeks. As our team reported previously, the following dosages of dexamethasone were used: 2.25 mg once daily for two weeks, 1.5 mg once daily for one week, and 0.75 mg once daily for one week. Following the end of the pharmacological treatment, all patients were subsequently monitored for an additional seven weeks. Regular phone calls to participants and their carers, as well as weekly visits for clinical evaluation and medication instructions, were used to keep tabs on treatment compliance. Poor therapeutic efficacy (poor absorption) was defined as the need to switch to surgery as a result of a decline in neurological status and/or an expansion of the hematoma volume, whereas good therapeutic efficacy (good absorption) was defined as an improvement in neurological status and/or a decreased or stable hematoma volume during the 12-week follow-up that did not require a change in therapy.

### Data collection and image acquisition

The following clinical and demographic data were retained from medical records: patient age, sex, history of head trauma, MGS–GCS scores, and history including hypertension, diabetes mellitus (DM), and use of anticoagulant and/or antiplatelet agents.

Brain CT scans taken before medication therapy were used for this study. Through the hospital's picture archiving and communication system (PACS), the imaging data from 141 patient brain CT scans (64-slice, GE Healthcare, Chicago, IL) were obtained and archived in the DICOM format. The CT scanning range encompassed the base of the skull to the cranium with a scan thickness of 5 mm, a detector width of 0.625 mm, a tube voltage of 120 kV, a tube current of 260 mAs, a matrix size of 512 × 512, and iterative reconstruction algorithm (ASiR-V with a weight percentage of 30%). The CT images were set with a window width of 80 HU and a window level of 35 HU.

### Image data processing and quantitative image analysis

Quantitative volumetric analyses and CTTAs were performed on the CT images. The following imaging characteristics were evaluated in this study: (a) hematoma laterality, left versus right; (b) hematoma site, unilateral versus bilateral; (c) hematoma volume; (d) midline shift; (e) maximal thickness of the hematoma; (f) hematoma density to brain parenchyma density ratio; and (g) hematoma texture parameters: singal min (minimum), singal max (maximum), singal mean, and singal standard deviation of the pixel distribution histograms; inhomogeneity, skewness, kurtosis, and entropy. For quantitative volumetric analysis, the hematoma regions of interest (ROI) were traced for each axial slice by two neurosurgeons (CG and XZ, both with eight years of experience in neurosurgery), who were blinded to chronic subdural hematoma patients’ conditions. The hematoma volume and CTTA histograms were generated by FireVoxel, a free medical image texture analysis and processing platform (https://firevoxel.org/). The hematoma volume and texture parameters from the two neurosurgeons were averaged.

**Figure 1:**
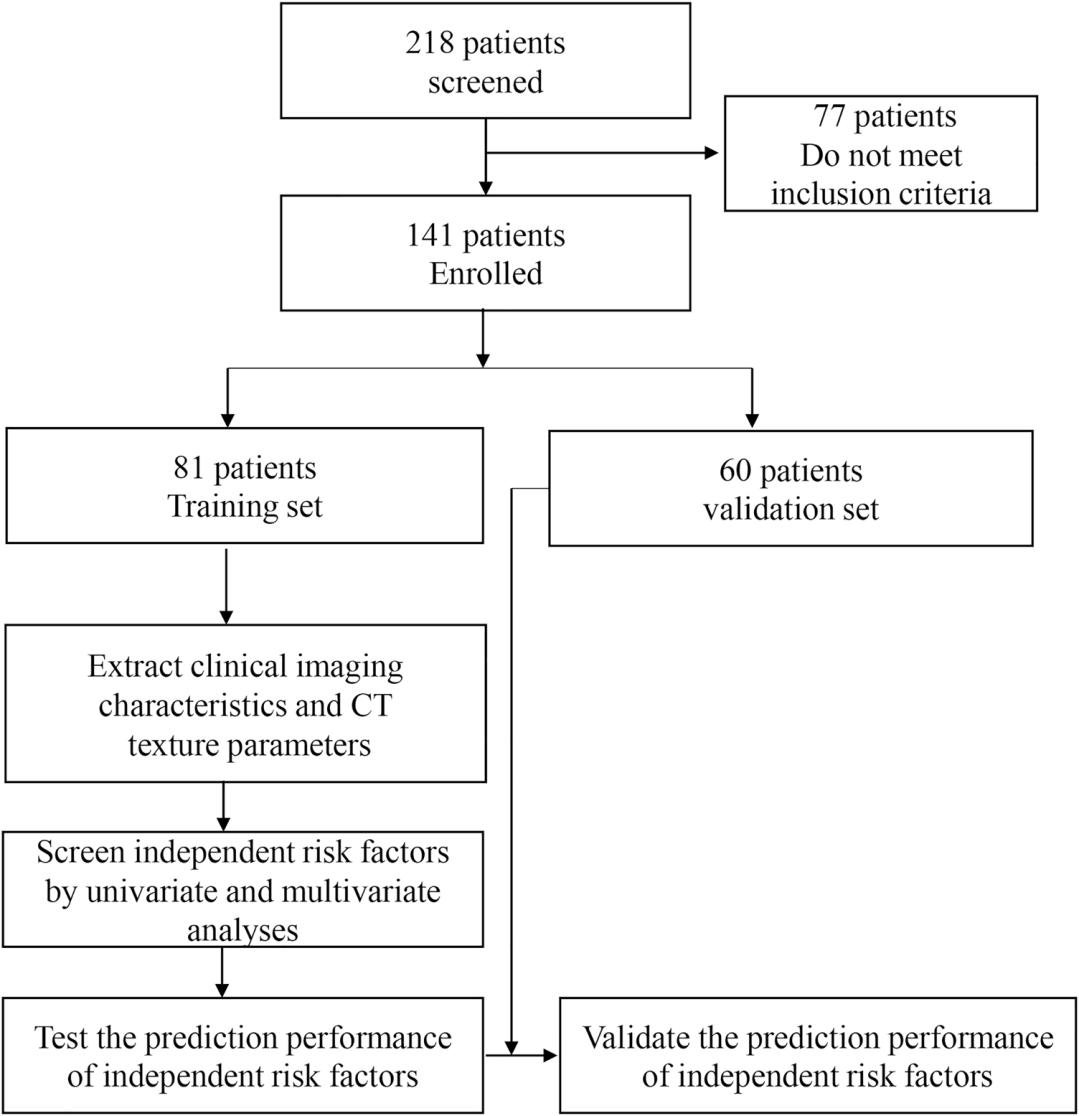
Flow chart of the study. CT, computed tomography.

The development and analysis process of this study are shown in Fig. 1.

### Statistical analysis

The IBM SPSS 24.0 software package (SPSS Inc., Chicago, IL, USA) was used to conduct all statistical analyses. Stratified sampling was used to separate the patients into a training set (60% of patients) and a validation set (40% of patients). First, univariate analysis was carried out to look for any potential relationships between the research variables and the therapeutic effectiveness of CSDH. For continuous variables, the variables were checked for normal distribution by the Shapiro‒Wilk test, and the unpaired Student's t-test (normal distribution) or Mann‒Whitney U test (nonnormal distribution) was employed to compare two independent groups. For categorical variables, Fisher’s exact and χ^2^ tests were applied. Multivariate stepwise logistic regression was conducted to control confounding factors and identify potential factors of poor absorption addressed by polytherapy for chronic subdural hematoma through the covariates (*p* < 0.05) screened by univariate analysis. Areas under the curve (AUC), sensitivity, specificity, and cutoff values, which were produced via ROC curve analysis, were employed in our study to calculate the potential predictive power of significant factors. The cutoff values for significantly different factors evaluated with the training set were applied to the validation set. Accuracy, sensitivity, and specificity were employed to establish the validity of key factors. All tests were two-tailed, and *p* < 0.05 was defined as significant.

### Ethical approval

The study was conducted in accordance with the Declaration of Helsinki (as revised in 2013). This study was approved by the Institutional Ethics Committee of Tianjin Medical University General Hospital.

### Informed consent

The requirement of written informed consent from participants was waived by the Institutional Ethics Committee of Tianjin Medical University General Hospital as the analysis of data was from a deidentified database.

## Results

**Figure 2:**
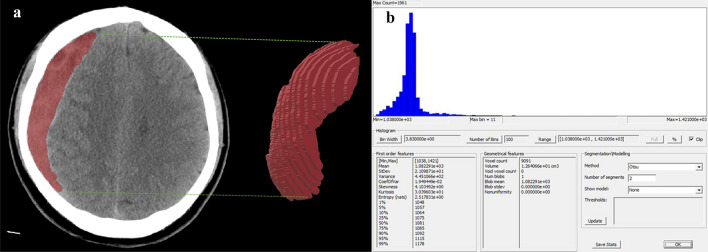
An example showing the results of delineating the ROI and analyzing the hematoma by FireVoxel software. (**a**) ROI delineated by neurosurgeons. (**b**) The image of measurements exported from FireVoxel. ROI, regions of interest.

The clinical characteristics of 141 patients with chronic subdural hematoma are summarized in Table 1. The patient population with CSDH contained 91 males and 50 females, with a mean age of 64.98 years at the time of drug treatment. In the training set, 63 patients were classified into the good absorption group, and 18 patients were classified into the poor absorption group. The texture parameters were obtained by FireVoxel, and an example is shown in Fig. 2.

**Table 1:** Clinical characteristics of patients with CSDH enrolled in the study.

Characteristic	Number of patients (%)
Total	141
Sex	
Male	91(64.5)
Female	50(35.5)
Age, mean ± SD (years)	
Male	69.09 ± 13.94
Female	60.87 ± 21.42
Traumatic brain injury history	
Yes	78(55.3)
No	63(44.7)
Hematoma site	
Unilateral	82(58.2)
Left, 46(56.1); Right, 36(43.9)	
Bilateral	59(41.8)

*CSDH* chronic subdural hematoma, *SD* standard deviation.

Tables 2 and 3 display the univariate and multivariate analyses of the relationships between the different research factors and the therapeutic efficacy of CSDH. The clinical and radiological characteristics of the good and poor absorption groups were compared. The results of the univariate examination of potential risk variables showed that no clinical characteristics were associated with the therapeutic efficacy of CSDH. From the radiographic factors, hematoma volume (*p* = 0.004), midline shift (*p* = 0.003), hematoma density to brain parenchyma density ratio (*p* = 0.014), signal min of the pixel distribution histograms (*p* < 0.001), signal max of the pixel distribution histograms (*p* < 0.001), signal mean of the pixel distribution histograms (*p* = 0.034), signal standard deviation of the pixel distribution histograms (*p* = 0.019), inhomogeneity (*p* = 0.046), kurtosis (*p* = 0.035), and entropy (*p* < 0.001) were significantly associated with the therapeutic efficacy of CSDH. The multivariate analysis with a logistic regression model identified the hematoma density to brain parenchyma density ratio (OR, 0.938; 95% CI 0.886–0.994; *p* = 0.029), signal min of the pixel distribution histograms (OR, 0.979; 95% CI 0.961–0.996; *p* = 0.019), signal standard deviation of the pixel distribution histograms (OR, 1.247; 95% CI 0.064–0.948; *p* = 0.042) and hematoma inhomogeneity (OR, 2.546; 95% CI 1.052–6.160; *p* = 0.038) as independent risk factors for the therapeutic efficacy of polytherapy for chronic subdural hematoma.

**Table 2:** Univariate analysis of factors for the outcome of CSDH patients based on polytherapy in the training set.

Factor	Good absorption group	Poor absorption group	*p*-value
Total	63	18	
Sex			
Male	50	16	0.566
Female	13	2	
Age (years)	62.27 ± 17.35	68.61 ± 8.40	0.752
Hypertension			
Yes	27	8	0.905
No	36	10	
Diabetes mellitus			
Yes	14	3	0.855
No	49	15	
Anti-coagulant/anti-platele			
Yes	8	3	0.965
No	55	15	
Traumatic brain injury history			
Yes	31	11	0.373
No	32	7	
MGS–GCS			
0	16	2	0.072
1	37	11	
2	10	5	
Time from the onset to receiving CT scans (days)	4.85 ± 1.90	5.40 ± 2.11	0.169
Hematoma site			
Unilateral	33	12	0.282
Bilateral	30	6	
Hematoma volume (ml)	59.25 ± 43.41	91.21 ± 29.49	0.004**
Midline shift (mm)	3.88 ± 3.94	7.19 ± 4.35	0.003**
Maximal thickness of hematoma (mm)	16.35 ± 7.26	18.99 ± 7.42	0.179
Hematoma density to brain parenchyma density ratio (%)	98.86 ± 39.46	73.65 ± 28.50	0.014*
Hematoma texture parameter			
Singal min of pixel distribution histogram	1030.00(1020.00, 1070.00)	925.00(544.30, 1040.00)	< 0.001***
Singal max of pixel distribution histogram	1330.00 (1250.00, 2250.00)	1495.00 (1358.00, 2490.00)	< 0.001***
Singal mean of pixel distribution histogram	1056.00 ± 12.53	1028.00 ± 102.00	0.034*
Singal median of pixel distribution histogram	1055.00 ± 13.54	1049.00 ± 11.10	0.143
Singal standard deviation of pixel distribution histogram	11.50 (8.74, 44.20)	12.75 (11.48, 471.00)	0.019*
Inhomogeneity	0.013 ± 0.001	0.057 ± 0.175	0.046*
Skewness	4.35 ± 4.10	2.86 ± 4.91	0.195
Kurtosis	39.20 (19.60, 402.00)	85.65 (52.23, 517.00)	0.035*
Entropy	2.46 ± 0.49	1.99 ± 0.43	< 0.001***

*CSDH* chronic subdural hematoma, *MGS–GCS* Markwalder grading scale–Glasgow Coma Scale.

**Table 3:** Univariate and multivariate analysis of factors for the outcome of CSDH patients based on polytherapy in the training set.

Factor	Univariate analysis, *p*-value	Multivariate analysis, *p*-value	OR	95% CI
Hematoma volume (ml)	0.004**	0.518	1.009	0.982–1.037
Midline shift (mm)	0.003**	0.304	1.113	0.907–1.367
Hematoma density to brain parenchyma density ratio (%)	0.014*	0.029*	0.938	0.886–0.994
Hematoma texture parameter				
Singal min of pixel distribution histogram	< 0.001***	0.019*	0.979	0.961–0.996
Singal max of pixel distribution histogram	< 0.001***	0.052	1.014	1.000–1.029
Singal mean of pixel distribution histogram	0.034*	0.050	1.186	1.000–1.406
Singal standard deviation of pixel distribution histogram	0.019*	0.042*	1.247	0.064–0.948
Inhomogeneity	0.046*	0.038*	2.546	1.052–6.160
Kurtosis	0.035*	0.698	0.997	0.982–1.012
Entropy	< 0.001***	0.504	4.572	0.053–395.744

*CSDH* chronic subdural hematoma, *OR* odds ratio, *CI* confidence interval.

**Figure 3:**
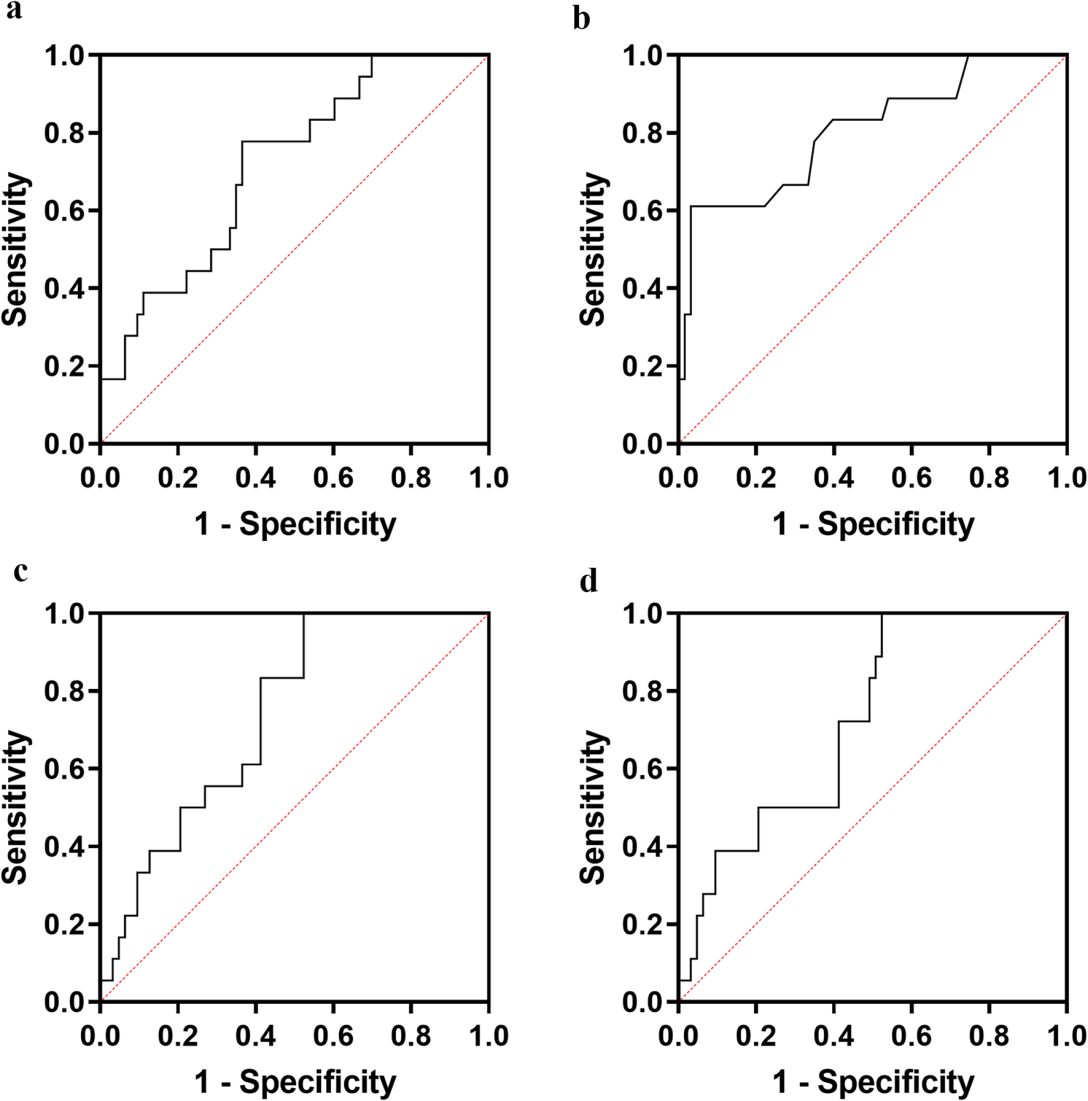
ROC analysis of the independent risk factors in the training data. Hematoma density to brain parenchyma density ratio (**a**), signal min of the pixel distribution histograms (**b**), signal standard deviation of the pixel distribution histograms (**c**), and hematoma inhomogeneity (**d**) had excellent predictive values for CSDH patients based on polytherapy. CSDH, chronic subdural hematoma; ROC, receiver operating characteristic.

ROC curves are shown in Fig. 3, and Table 4 shows the AUC, the optimal threshold, and the corresponding accuracy of the independent risk factors for predicting the therapeutic efficacy of polytherapy for CSDH in the training data. With the hematoma density to brain parenchyma density ratio, the AUC was 0.716, with a sensitivity of 0.778, a specificity of 0.635, and a cutoff value of 84.130. The AUCs of the CT texture parameters signal min, signal standard deviation of the pixel distribution histograms, and inhomogeneity were 0.806, 0.737, and 0.723, respectively, with sensitivities of 0.611, 0.833, and 0.833, respectively, specificities of 0.968, 0.587, and 0.508, and cutoff values of 979.500, 10.330, and 0.011, respectively.

**Table 4:** ROC curve analysis of factors for the outcome of CSDH patients based on polytherapy in the training set.

Factor	AUC	Sensitivity	Specificity	Cut-off	*p*-value
Hematoma density to brain parenchyma density ratio (%)	0.716	0.778	0.635	84.130	0.005**
Singal min of pixel distribution histogram	0.806	0.611	0.968	979.500	< 0.001***
Singal standard deviation of pixel distribution histogram	0.737	0.833	0.587	12.330	0.002**
Inhomogeneity	0.723	0.833	0.508	0.011	0.004**

*ROC* receiver operating characteristic, *CSDH* chronic subdural hematoma.

Table 5 shows the accuracy of the independent factors’ predictive performance in the validation set. The accuracy, sensitivity, and specificity for the hematoma density to brain parenchyma density ratio were 0.869, 0.700, and 0.640, respectively. The accuracies of the computed tomography texture parameters signal min, signal standard deviation of the pixel distribution histograms, and inhomogeneity were 0.952, 0.816, and 0.816, respectively, with sensitivities of 0.700, 0.900, and 0.900, respectively, and specificities of 0.825, 0.530 and 0.520, respectively.

**Table 5:** Accuracy of related factors for the outcome of CSDH patients based on polytherapy in the validation set.

Factor	Sensitivity	Specificity	Accuracy
Hematoma density to brain parenchyma density ratio (%)	0.700	0.640	0.869
Singal min of pixel distribution histogram	0.700	0.825	0.952
Singal standard deviation of pixel distribution histogram	0.900	0.530	0.816
Inhomogeneity	0.900	0.520	0.816

*CSDH* chronic subdural hematoma.

## Discussion

Our study analyzed the clinical imaging features and computed tomography texture parameters of chronic subdural hematoma patients receiving polytherapy to predict their treatment efficacy. We found that the hematoma density to brain parenchyma density ratio, min of pixel distribution histogram, standard deviation of pixel distribution histogram, and inhomogeneity can predict the thermal efficiency of polytherapy, which has good sensitivity and specificity. Our previous research confirmed that polytherapy can effectively promote the absorption of chronic subdural hematoma. However, some patients have poor efficacy with this treatment. Our study found four factors that can predict the prognosis, providing a basis for the treatment plan for CSDH patients.

In this study, we used univariate and multivariate analyses, and the results showed that the clinical factors of patients with CSDH were not correlated with the prognosis.

Despite numerous investigations that looked into the pathophysiology of the diseases, which included bridging vein avulsion hemorrhage, increased osmolality, hematoma capsule bleeding, and local hyperfibrinolysis, the pathogenesis and breakdown mechanism of CSDH are still poorly understood. In hematomas, various levels of inflammatory cytokines and vascular endothelial growth factor (VEGF) are produced. This induces the creation of juvenile blood vessels on the capsule, the death of vascular endothelial cells, and the opening of gap junctions. A hematoma eventually develops as a result of the ongoing chemical leakage caused by the increasing permeability. In this study, the univariate analysis showed that hematoma volume, midline shift, and hematoma density to brain parenchyma density ratio were associated with the prognosis. Due to controlling for confounding factors, the influence of these factors was reduced in the multivariate analysis, and the ratio of hematoma density to brain parenchymal density was closely related to the prognosis. The subdural hematoma density was classified into four groups based on the computed tomography findings: low density, isodense, hyperdense, and mixed density^25^. However, this study identified the hematoma density to brain parenchyma density ratio as an independent factor, further reducing confounding factors. We found that when the ratio of hematoma density to brain parenchymal density was much less than 100, the hematoma absorption effect was poor, which may be related to the composition of the hematoma. During this time, the hematoma of CSDH patients is filled with blood degradation products and plasma, which are constantly reabsorbed. This means that as the hematoma matures, the new capillaries in the membrane become less fragile and leak; therefore, relatively “stationary” hematomas may not be easily affected by atorvastatin and dexamethasone.

Texture analysis has been a research hotspot in imaging in recent years. Clinicians often make judgments based on theoretical knowledge and clinical experience when diagnosing various diseases. Texture analysis quantitatively analyzes the spatial relationships, pixel relationships, and grayscale distribution relationships of images, indirectly reflecting the pathological characteristics of lesions^26^ and not influenced by subjective factors.

However, the majority of the interest in quantitative imaging biomarkers has been in assessing the therapeutic response of tumors. Recently, many studies have also applied texture parameters to other issues. Shan et al. discovered that compared to other regularly used intracranial pressure evaluation methods, the first-order texture analysis method performed better in determining intracranial pressure values^27^. To predict cerebral vasospasm, delayed cerebral ischemia, and functional outcome in aneurysmal subarachnoid hemorrhage, Kanazawa et al. identified CT texture-related metrics (mean CT value, entropy, skewness, and kurtosis) in the early postictal state^28^. To forecast early hematoma expansion, Shen et al. examined the picture texture (mean gray-level intensity, variance, and uniformity of the ROI) of head CT images^29^.

Heterogeneity reflects the complexity and irregularity of the internal structure of an organization, typically associated with entropy (irregularity), kurtosis (magnetism of pixel distribution), skewness (skewness of pixel distribution), and signal value (min, max, mean, median, and standard deviation) of the pixel distribution histograms. In general, a higher entropy, a higher standard deviation of the pixel distribution histogram, a higher kurtosis, and a lower skewness represent increased inhomogeneity. Kanazawa et al. discovered that preoperative hematoma entropy exhibited an inclination to be linked with the recurrence of both symptomatic and potential chronic subdural hematoma based on univariate analysis. However, this association did not hold in the multivariate analysis due to the presence of confounding factors. In our study, after controlling for confounding factors, the min, standard deviation of the pixel distribution histogram, and inhomogeneity were identified as independent factors that can predict the prognosis. The minimum and standard deviation of the pixel distribution histogram can also reflect hematoma heterogeneity. Therefore, overall, the greater the hematoma heterogeneity is, the worse the prognosis.

Surgery with subdural drainage is the mainstay treatment for chronic subdural hematoma. However, owing to the potentially severe complications, a recurrence risk as high as 30%, and the elevated moral rates within this susceptible patient cohort^7–10^, the pursuit of secure and efficacious nonsurgical treatments is paramount. Our previous research showed the safety and effectiveness of atorvastatin for treating patients with chronic subdural hematoma^17,18^. Based on this research, we further demonstrated that ATO combined with DXM was more effective than ATO alone, as it successfully reduced hematoma size and improved neurological function in patients with chronic subdural hematoma^21^. Nonetheless, a subset of patients failed to derive benefits from polytherapy. For individuals lacking the capacity for hematoma self-absorption, undergoing polytherapy could pose risks and yield minimal rewards. Hence, identifying prognostic factors is imperative for CSDH patients undergoing polytherapy. Our study identified relevant factors that can predict the self-absorption capacity of hematoma in CSDH patients receiving polytherapy by using clinical imaging characteristics in conjunction with computed tomography texture analysis. This research result will help us determine which type of CSDH patients are more suitable for polytherapy, further improving the safety of polytherapy for chronic subdural hematoma.

Several limitations are associated with this study. First, it is a retrospective single-center study that is potentially susceptible to selection bias. Next, recent research reported that endoscopic findings were related to chronic subdural hematoma recurrence^30^, which might be an independent risk factor in our study. However, our research focused on patients receiving conservative treatment, and we were unable to obtain data under endoscopic surgery. Additionally, the study’s application of ROI carries various limitations. Inevitable bias could arise due to manual ROI delineation and visual positioning. Incorporating automated ROI segmentation would enhance objectivity in measurements. In the utilization of first-order CT texture histogram analysis, pixel locations and the spatial interplay between gray values are not considered, thereby restricting the information yielded, and higher-order texture features were not analyzed. Last, we lack external validation of the study because it was based on a single center. To confirm our findings, more centers need to enrol.

## Conclusions

The ratio of hematoma density to brain parenchymal density is closely related to the prognosis of CSDH patients receiving polytherapy. In addition to them, min, standard deviation of pixel distribution histogram, and inhomogeneity are also identified as independent factors that can predict the prognosis by using computed tomography texture analysis. This research outcome will aid us in identifying the type of CSDH patients that are better suited for polytherapy, thereby enhancing the safety profile of this therapeutic approach for chronic subdural hematoma.

### Supplementary Information

Supplementary Information.

## Data Availability

Data is available from the corresponding author upon reasonable request.
